# Additions to Dr. Potter’s Paper

**Published:** 1895-02

**Authors:** William H. Potter

**Affiliations:** Boston


ADDITIONS TO DR. POTTER'S PAPER.

TO THE EDITOR:

SIR,—In my article entitled "Some Details as to the Care of Instruments," appearing in the December, 1894, number of the INTERNATIONAL DENTAL JOURNAL, I make the statement that carbonate of soda was substituted for potassic hydrate in my system of sterilizing because cheaper and equally effective. I have, however, found specimens of carbonate of soda which were not satisfactory, because they did not keep steel instruments from rusting while being sterilized. And I have returned to my original plan of using a potassic hydrate solution for the prevention of rust. I use two or three drachms of the liquor potassæ of the Pharmacopœia in a pint of water. Instead of using a zinc tray inside of the sterilizer, as described in my article, I am now using a porcelain enamelled iron dish such as is made for cooking purposes. This porcelain enamelled dish I find keeps the instruments in better condition than a metallic tray. For thorough sterilization instruments should be kept at 100° centigrade, moist heat, for over thirty minutes. It is my practice to leave instruments in the sterilizer about one hour.

WILLIAM H. POTTER, D.M.D.

BOSTON, January 16, 1895.

